# Asymmetry of Chromosome Replichores Renders the DNA Translocase Activity of FtsK Essential for Cell Division and Cell Shape Maintenance in *Escherichia coli*

**DOI:** 10.1371/journal.pgen.1000288

**Published:** 2008-12-05

**Authors:** Christian Lesterlin, Carine Pages, Nelly Dubarry, Santanu Dasgupta, François Cornet

**Affiliations:** 1 Laboratoire de Microbiologie et de Génétique Moléculaire, Centre National de la Recherche Scientifique, Toulouse, France; 2 Université de Toulouse, Université Paul Sabatier, Toulouse, France; 3 Centre de Génétique Moléculaire, Centre National de la Recherche Scientifique, Gif-sur-Yvette, France; 4 Department of Cell and Molecular Biology, Uppsala University, Uppsala, Sweden; Stanford University, United States of America

## Abstract

Bacterial chromosomes are organised as two replichores of opposite polarity that coincide with the replication arms from the *ori* to the *ter* region. Here, we investigated the effects of asymmetry in replichore organisation in *Escherichia coli*. We show that large chromosome inversions from the terminal junction of the replichores disturb the ongoing post-replicative events, resulting in inhibition of both cell division and cell elongation. This is accompanied by alterations of the segregation pattern of loci located at the inversion endpoints, particularly of the new replichore junction. None of these defects is suppressed by restoration of termination of replication opposite *oriC*, indicating that they are more likely due to the asymmetry of replichore polarity than to asymmetric replication. Strikingly, DNA translocation by FtsK, which processes the terminal junction of the replichores during cell division, becomes essential in inversion-carrying strains. Inactivation of the FtsK translocation activity leads to aberrant cell morphology, strongly suggesting that it controls membrane synthesis at the division septum. Our results reveal that FtsK mediates a reciprocal control between processing of the replichore polarity junction and cell division.

## Author Summary

In most bacteria, chromosomes consist of a single replication unit. Replication initiates at a single *ori* and terminates in the diametrically opposite zone, *ter*, defining two replication arms. These are also called replichores to account for base composition and sequence motif polarity biases from *ori* to *ter*. This organisation as symmetrical replichores is conserved in bacteria. Here, we have investigated the effect of replichore asymmetry in *E. coli*. We show that it blocks the cell cycle at a step posterior to chromosome replication. This phenotype is not corrected by restoration of the termination of replication opposite *ori*, strongly suggesting that it is due to the asymmetry of replichore polarity. FtsK, a DNA-translocase associated with the division septum that processes the terminal junction of replichore polarity, is essential for growth and for the controlled blockage of cell growth in cells with asymmetric replichores. These results reveal a reciprocal control between the processing of the terminal junction of the replichores by FtsK and the progression of the cell cycle.

## Introduction

Cell proliferation involves coordination between replication of the genome, segregation of sister chromosomes to daughter cells and cell division. In bacteria, these processes are not necessarily sequential but can overlap: segregation begins during the replicative C period (S phase), continues during the post-replication D period (G2/M) and may last until constriction of the division septum.

**Figure 1:**
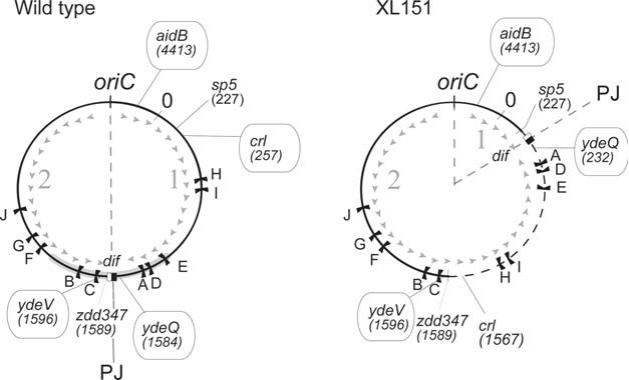
Map of the wild type and XL151 chromosomes. The positions of Inv(*dif-sp5*) inversion endpoints (the *sp5* and the *zdd347* loci) and the loci used for *parS* insertions (*aidB, crl, ydeQ* and *ydeV*) are indicated. Coordinates are in kb. The black flags are the replication terminators (Ter sites with corresponding letter from A to J). The grey arrowheads and numbers represent the polarity (orientation of KOPS motifs) of replichore 1 and 2, respectively, and the grey heavy line the Ter macrodomain. PJ: polarity junction that contains the *dif* site (the black and white square); *oriC*: origin of replication.

In *Escherichia coli*, chromosome replication initiates at a single origin, *oriC*, and progresses bidirectionally to the termination region, *ter*. The *ter* region is flanked by multiple replication terminators (Ter sites in Figure 1), which when bound by the Tus protein, stop replication forks in a polar manner [1]. This replication mode defines two replication arms called replichores, characterised by the skewed orientation of numerous sequence elements from *oriC* to *ter* [2]. One of these, an octameric motif termed KOPS, is involved in chromosome segregation (see below) and defines the terminal junction of replichore polarity (PJ in Figure 1).

The segregation process follows replication in a sequential manner so that chromosome sequences are segregated in the order of their position on the replichores 3–5. Following replication, newly replicated chromosome loci remain co-localised, or cohered, for a period, which varies according to growth conditions and locus position, then migrate from mid-cell to the quarter-cell positions [4], [6]–[13]. Opposite replichores tend to segregate to defined positions around the cell quarters so that the preferred segregation pattern yields a left-right-left-right organisation of the replichores in the daughter cells [5],[10],[14]. Lastly, the chromosome is organised into four 0,5 to 1 Mb macrodomains termed Ori, Right, Left and Ter, composed of sequences displaying similar positioning and segregation timing [3],[15],[16].

The *ter* region is the last segregated [3],[7],[9], and hosts the post-replicative events required for the physical separation of sister chromosomes [17]. These processes are concomitant with the assembly and constriction of the division septum and are controlled by the septum-associated FtsK translocase [18]–[21]. FtsK is a multifunctional protein [22]. Its N-terminal domain localizes FtsK to the division septum and is essential for cell division [23]–[25]. Its C-terminal domain carries an ATP-dependent DNA translocase activity [26],[27]. FtsK translocation is oriented by recognition of the KOPS DNA motifs [28],[29]. The KOPS orientation is highly biased along the replichores and changes at the *dif* site, dedicated to resolution of chromosome dimers. FtsK thus reaches *dif* and, when required, activates XerCD/*dif* recombination to resolve dimeric chromosomes [26],[30],[31]. FtsK is also thought to assist the removal of catenation links that persist between sister chromosomes after replication. It activates TopoIV, the major decatenase [21], and promotes decatenation by XerCD/*dif* recombination in certain conditions [32].

We have previously reported that large chromosome inversions with a fixed endpoint at the *dif* position (the terminal polarity junction, PJ) and a second endpoint outside the Ter macrodomain provoke a specific cell-cycle defect in *E. coli* [33]. Strains carrying these inversions fail to grow at high temperatures or in rich medium. In both permissive and non-permissive growth conditions, cells undergoing division are over-represented suggesting that division is delayed or blocked due to the inversion. Here we have analysed a representative of this set of strains in further details. We show that the inversion delays or block the cell cycle in late D period. This effect is not due to the imbalance of replication arms since restoration of replication termination opposite *oriC* does not suppress the D period blockage. Chromosome loci at the edge of the inversion show defects in their intracellular localisation, particularly the displaced PJ. Most interestingly, translocation by FtsK becomes essential in the inversion-carrying strain and its inactivation leads to defects in peptidoglycan synthesis.

## Results

### Inversions Affect Late Steps of the Cell Cycle

**Figure 2:**
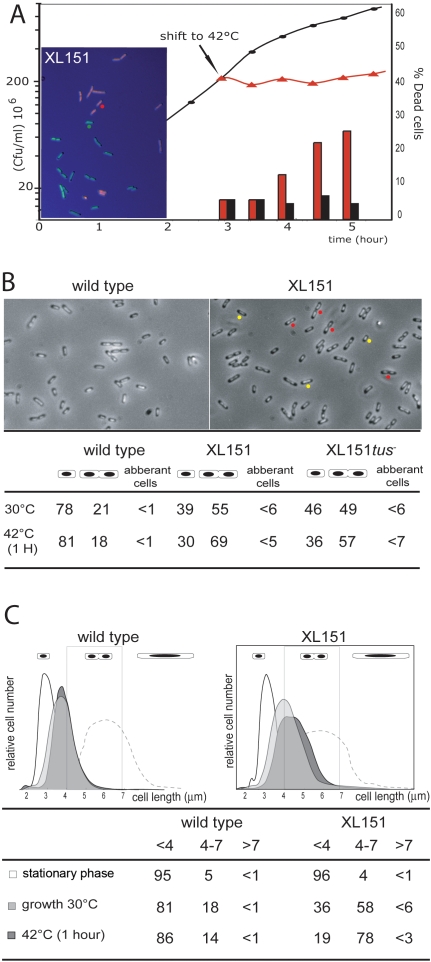
Phenotype of the XL151 strain. A) XL151 was grown in M9 broth at 30°C (permissive conditions; closed circles) and growth was followed by plating in permissive conditions (cfu, left scale). At the indicated time, one half of the culture was shifted to 42°C (non-permissive conditions; red triangles). The fraction of dead cells was followed in both culture using live and dead staining (300 cells per count; right scale; black bars: permissive conditions; red bars: non-permissive conditions). A micrograph of XL151 stained with Live/dead staining (Materials and Methods) after incubation 5 hours in non-permissive conditions is shown (dead cells: red; Live cells: green; examples are indicated with the red and green dots, respectively). B) Top: micrographs of wt (left) and XL151 (right) after 1 hour at 42°C (non-permissive conditions). Example of cells counted as single (yellow dots) and double cells (red dots) are shown. Bottom: cells with one nucleoid (single cells), cells with two apparently segregated nucleoids (harbouring or not harbouring a division septum; double cells) and abnormal cells (filaments and chains) were counted in permissive conditions (30°C) and after 1 hour in non permissive conditions (42°C) (300 cells per count). C) The cell size distribution of wt (left) and XL151 (right) was determined by flow cytometry in different conditions: after overnight growth in permissive conditions (stationary phase, white); in exponential growth in permissive conditions (growth 30°C, pale grey); after one hour incubation in non-permissive conditions (42°C-1H, dark grey) and 1 hour after addition of cephalexin in permissive conditions (dash line). Size is in mm (x-axis). Quantifications of the fractions of cell under 4 µm, from 4 µm to 7 µm and over 7 µm are presented (see also Table S1).

Strain XL151, which carries the Inv(*dif-sp5*) inversion, was chosen as a representative strain carrying a large inversion from the terminal junction of replichore polarity (PJ) (Figure 1). It carries the *dif* site at the new PJ (Figure 1) and is thus proficient for resolution of chromosome dimers. This strain displays sensitivity to high temperatures (Figure 2A) and rich medium characteristic of such strains [33]. In permissive growth conditions, XL151 displays a 92 min doubling time compared with 64 min for its parent non-inverted strain (wt) (Table 1). This growth defect is not accompanied by the appearance of large numbers of cells that are dead, filamented, in chains or aberrantly shaped (Figure 2A and B). Cells with two apparently segregated nucleoids and, most frequently, a constricting division septum (herein called double-cells, examples are tagged with red dots in Figure 2B) are over-represented in XL151 populations growing under permissive conditions (∼55% compare to 21% for wt; Figure 2A). These data suggest that the main effect of the inversion on cell growth is to delay cell division.

**Table 1:** Cell cycle parameters of wt and XL151 strains.

Relevant Genotype	Doubling time(1)	C+D Period(2)	C period(1)	D period(2)
wild type 30°C	64 +/− 2	90	43.8 +/− 1,4	∼46
wild type 42°C	56 +/− 2	68	40 +/− 5 (3)	∼28
XL151 30°C	92 +/− 3	123	64 +/− 3,5	∼59
XL151 42°C	NA	NA	55 +/− 5 (3)	NA
XL151 *tus* 30°C	86 +/− 3	111	48.2 +/− 0,8	∼63

Times are in minutes. Data are the mean of at least 3 independent experiments.

(1) data measured.

(2) data calculated using the Helmstetter's model (origin per cell = 2^(C+D)/t^; Helmstetter, 1996). Note that this model originally designed for *E. coli* B/r has been validated for K12 strains grown in these conditions [56]. Details of origin per cell calculation are in TableS1.

(3) Data from flow cytometry experiments.

NA: not applicable.

After a shift to non-permissive conditions, cells rapidly stop growing and the fraction of double-cells increased (69% compare to 18% for wt after a 1 hour shift; Figure 2A and B). Notably, the percentage of aberrant cells remains low (Figure 2B) and the percentage of dead cells starts to rise only after 1 hour (Figure 2A). Examination of XL151 after long incubation in non-permissive conditions (5 hours in Figure 2A) show that most dead cells are dividing cells, suggesting that cells die at the time of division. Some small dead cells can be detected which may originate from abortive divisions. Taken together, these results suggest that the primary effect of the shift to non-permissive conditions is to block cell growth and division concomitantly.

These observations were confirmed by analysis of cell size distribution of XL151 by flow cytometry (Materials and methods; Figure 2C). Non-growing (stationary phase) XL151 and wt display similar cell size. However, double cells (4 to 7 µm) are over-represented in XL151 populations in either permissive or non-permissive conditions. Under all conditions, aberrant cells (>7 µm) represent no more than few percent of the population. This phenotype is not specific to the Inv(*dif*-*sp5*) inversion since other strains carrying interdomain inversions from the terminal PJ display the same behaviour (for example the Inv(*dif*-*sp39*) strain in Table S1). Nor is it specific to inversions from the terminal PJ since strains carrying inversions within the Ter macrodomain from the *dif* position do not show over-representation of double-cells (the Inv(*dif-ydfE*) strain in Table S1).

### Inversions Extend Both the C and D Periods

**Figure 3:**
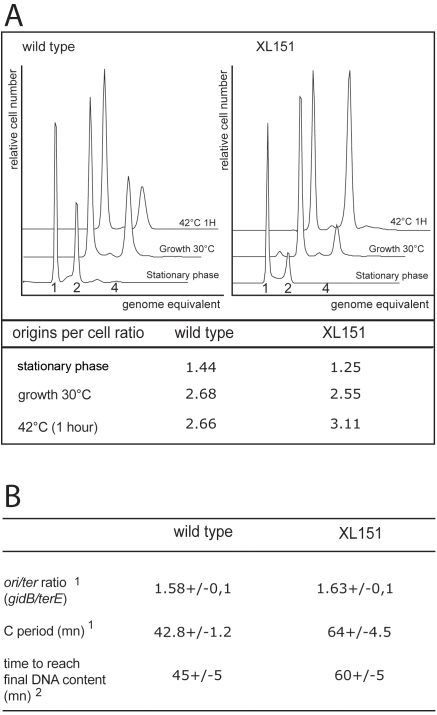
Replication in wild type and XL151. A) DNA content distribution of the wt (left) and XL151 (right) strains determined by flow cytometry after rifampicin/cephalexin run out (Material and methods) in stationary phase, exponential growth in permissive conditions (growth 30°C) and 1 hour in non-permissive conditions (42°C 1h). Chromosome equivalent are given for the different peaks. The average numbers of replication origins per cell in the three growth conditions is given below the graphs (see also Table S2). B) The C period of wt and XL151 cells growing under permissive conditions was measured using two techniques. ^1^the *ori/ter* ratio was determined by quantitative Southern blot hybridisation and the corresponding C period duration calculated. Data are the mean of three independent experiments with standard deviations (see Figure S1 for details). ^2^Data from flow cytometry analysis of the DNA content per cell after rifampicin treatment (Material and methods and Figure S1).

To gain insight into the cell cycle of XL151, we first analysed the timing of replication initiation using flow cytometry of cells in which initiation of replication and cell division had been blocked with rifampicin and cephalexin (Materials and methods; Figure 3A and Table S2). In XL151, the proportion of cells with an odd number of origins remains as low as in wt (<5%), indicating that initiation of replication is still synchronous. In addition, both XL151 and wt cells carry 2 or 4 origins showing that the inversion does not cause under- or over-initiation of replication and keeps initiation limited to once per generation (Figure 3A). The doubling time and the average number of origins per cell allow calculation of the timing of initiation and the C+D period (Materials and methods) [34]. The results presented in Table 1 show that the inversion provokes a 36% increase in C+D (123 min for XL151 compared with 90 min for the wt), which is consistent with the observed 43% increase in generation time. The XL151 cell cycle is thus affected at a step subsequent to the initiation of replication. Data obtained for another strain (Inv(*dif-sp39*); Table S2), with a similar inversion, led to the same conclusion.

The C period is expected to be longer in XL151 than in wt since Inv(*dif-sp5*) displaces replication terminators and extends one replication arms (Figure 1). To estimate the C period, the *ori/ter* ratio was measured by southern blot hybridisation (Figure 3B; Figure S1A) and by flow cytometry of rifampicin-treated cells (Figure 3B; Figure S1B and C). These two experiments gave consistent measures of the C period of ∼43 min for wt cells and ∼64 min for XL151 (Figure 3B; Table 1). This 49% increase of the C period due to the inversion is slightly weaker than expected from the 60% larger size of replichore 2 (3,687 Mb instead of 2,3 Mb for wt, Figure 1). This observation is consistent with previous work in equivalent strains [35],[36]. Consistently, we repeatedly observed a slightly higher copy number for the *ydeQ* and TerA loci when compared to the TerE locus in XL151 in southern blot hybridisations (not shown).

These data allow us to draw a general scheme of the cell cycle in wt and XL151 (Table 1; Figure S2), showing a significantly lengthened D period in XL151 (∼59 min compared with ∼46 min in wt). This lengthening of the post-replicative D period postpones cell division and is consistent with the over-representation of double-cells in XL151 cultures.

### Non-Permissive Conditions Block Both Cell Division and Elongation

Results presented in Figure 2 suggest that the blockage provoked by non-permissive conditions in XL151 occurs at the same step that is delayed in permissive conditions. To verify this conclusion, we analysed replication by flow cytometry after a shift to 42°C. The results show that non-permissive conditions do not induce asynchrony or uncontrolled over-initiation since the vast majority of cells contain 2 or 4 origins after 1 hour at 42°C (Figure 3A; Table 1). The absence of cells with 1 origin indicates that the growth arrest is not due to blockage of replication initiation (Figure 3A). Flow cytometry of rifampicin-treated cells shows that replication is complete by ∼55 min after the temperature shift (Figure S1C). We conclude that ongoing replication cycles proceed normally to their end under non-permissive conditions. This confirms that XL151 cells are blocked during the D period in non-permissive conditions.

Notably, a shift of growing XL151 to non-permissive conditions does not lead to massive production of cells longer than the normal cell size at division. Indeed, whereas double cells accumulate, only 5.5% of the cells are above 7 µm length 1 hour after a shift to 42°C (Figure 2B and C). For comparison, growing wt or XL151 treated with cephalexin for 1 hour continue to elongate so that most of the cells are longer than double-cells (Figure 2C, dashed line). We conclude that XL151 cells blocked in D period stop elongating. The modest increase in mean cell size of XL151 compared to wt in permissive conditions suggests that cell growth is also inhibited during the prolonged D period in permissive conditions (Figure 2C).

### Loose Intracellular Positioning of the Inversion Endpoints

**Figure 4:**
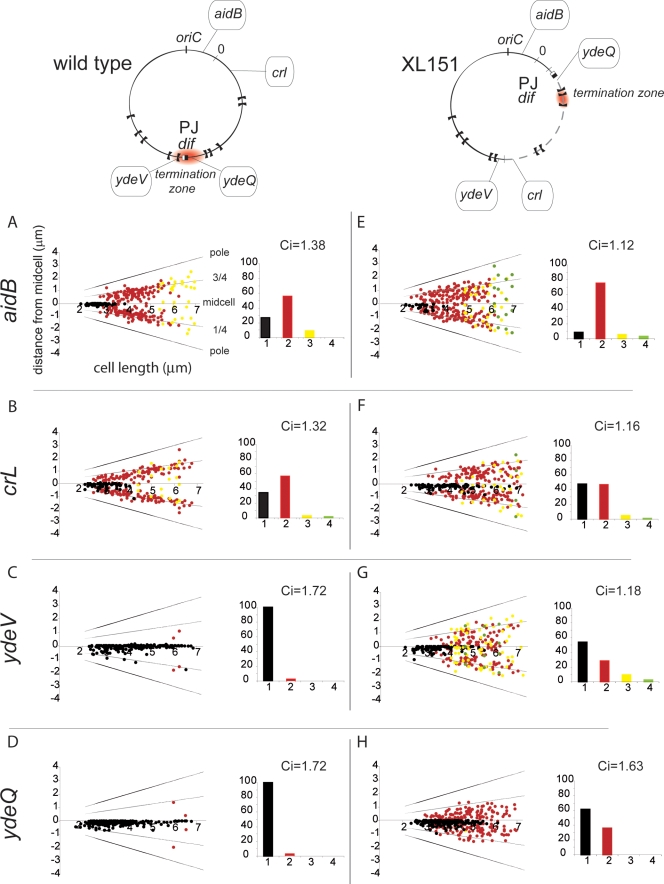
Chromosome segregation patterns in wild type and XL151. Top: representation of the wt and XL151 chromosomes with the replichore polarity junction (PJ), the termination zone (red zone) and the positions of *oriC*, *dif* and the *parS* insertions indicated. A to H: Intracellular localisation of the different *parS* insertions (indicated on the left) in wt (A to D) and XL151 (E to F). The dot plots represent the intracellular positioning of *parS* foci along the long axis of the cell (Y-axis with the cell poles, mid-cell and quarter positions indicated) as a function of cell length (X-axis) . Each plot represents data from 300 cells. Cell with one, two, three and four foci are respectively indicated in black, red, yellow and green. The histograms represent the proportion of cells with 1 focus (black), 2 (red), 3 (yellow) or 4 (green) foci. The Co-localisation Index (Ci) is obtained by dividing the mean locus copy number by the mean number of distinguishable fluorescent foci.

Results presented above show that an alteration of replichore polarity affects the post-replicative steps of the cell cycle, including cell division. We thus reasoned that this may be mediated by segregation defects. To analyse chromosome segregation in XL151, we used the Δ30ParB-GFP/*parS* system that enables the intracellular positions of loci into which the *parS* site is inserted to be determined by direct visualisation [4],[7]. We used *parS* insertions in *crl*, *ydeV* and *ydeQ* that mark the inversion endpoints, and in *aidB*, located close to *oriC* (Figure 1; Materials and methods). Figure 4 shows the fluorescent-foci positions as a function of cell length, the proportion of cells with 1, 2, 3 or 4 distinguishable foci (histograms) and the index of colocalisation (Ci) that reflects the time interval between replication and segregation of a locus (a Ci of 1 indicates immediate separation after replication; see Materials and methods and Table S3).

In the conditions used (64 min doubling time for the wt), sister origin region (*aidB*) and replichore 1 locus (*crl*) separate after a moderate period of colocalisation time (Ci = 1,38 and 1.32, respectively) and migrate from mid-cell to the quarter-cell positions while sister *ter* loci (*ydeQ* and *ydeV*) stay colocalised for a longer time (Ci = 1,72), remaining at mid-cell until division (Figure 4A to D). These observations are consistent with previous reports [4], [7], [9]–[11],[13]. The very small fraction of cells with more than one focus of *ydeV* and *ydeQ* further shows that the immediate vicinity of *dif* is the last separated zone of sister chromosomes [7],[9].

In XL151, we observed that the positioning of loci is generally looser than in the wt; localisation between the mid- and quarter-cell positions is more frequently observed (Figure 4E, F, G and H, compare to A, B, C and D, respectively). In XL151, *aidB* remains an *ori*-proximal loci, *ydeV* and *crl* are now at the two-thirds point of replichore 1 and *ydeQ* is at the new junction of replichore polarity (PJ) (Figure 1). Segregation of the *aidB* locus is the least affected, showing that the inversion does not provoke a significant mispositioning of the origin (Figure 4E). The frequency of cells with more than one *aidB* focus rises as a consequence of the postponement to division in XL151 compared to wt. Inv(*dif-sp5*) does not displace the *ydeV* locus on the chromosome but changes both its distance from the PJ and the timing of its replication with respect to termination. This is accompanied by a dramatic modification of its segregation pattern and a shorter colocalisation time (compare Figure 4C and G). The *crl* locus segregates in a manner similar to *ydeV* in accord with its new position (Figure 4F and G). The *ydeQ* locus remains at the PJ (Figure 1) and segregates in some respects as a terminal locus (Figure 4H). Most foci are in the vicinity of the septum and the colocalisation index remains high (Ci = 1.63). However, numerous cells with 2 foci appear (∼39% of total cells), mostly those that are dividing or about to divide (87% in cells from 4 to 7 µm). We conclude that the Inv(*dif-sp5*) inversion provokes a looser positioning of all tested loci and an early separation of the *ydeQ* locus with respect to cell division which may be responsible for or linked to the prolonged D period in XL151.

### Restoring Termination Opposite *oriC*

**Figure 5:**
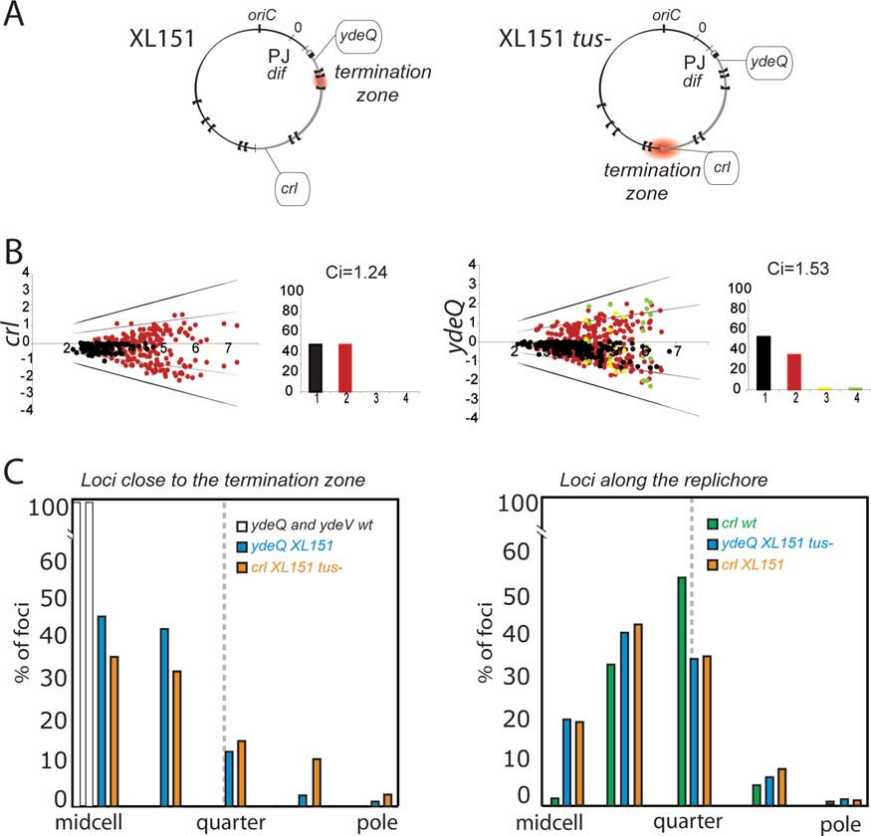
Effect of the restoration of termination opposite *oriC*. A) representation of the XL151 and the XL151 *tus*- chromosome with the replichore polarity junction (PJ), the termination zone (red zone) and the positions of *oriC*, *dif* and the *parS* insertion used indicated. B) intracellular positioning of the *crl* (left) and *ydeQ* (right) loci in XL151 *tus*-. same legend as Figure 4. C) Intracellular positioning of the different loci as a function of their position with respect to the replication termination zone. Cells were divided in 5 successive intervals from mid-cell to the pole and the fraction of foci in each interval is reported. Left: loci close to the termination zone. Cells with 2 foci are reported except for the *ydeQ* and *ydeV* loci for which cells with 1 focus are plotted. Right: loci far from the termination zone. Only cells with two foci are ploted. Data are from Figure 4 and 5B. The dashed grey line indicates the quarter position.

To analyse the role of the asymmetry of the replication arms in XL151, the *tus* gene was mutated to inactivate Ter sites. The resulting strain grows with a 86 min doubling time, intermediate between wt and XL151 (Table 1). The C period is ∼48 mn, consistent with the restoration of termination opposite *oriC* (Figure 5A; Table 1; Figure S1). However, the *tus* mutation did not suppress all effects of Inv(*dif-sp5*): double-cells are over-represented in permissive and non-permissive conditions (Figure 2B), the D period is still extended compared to wt (Table 1) and colony formation in non-permissive condition is not restored (not shown). The same partial effect of Tus inactivation has already been reported in equivalent strains [33]. The delay or blockage of the D period in XL151 is thus not attributable to the asymmetry of replication.

We next observed the effects of Tus inactivation on segregation of the *crl* and *ydeQ* loci in XL151 (Figure 5). The *crl* locus is now in the termination zone. It nevertheless does not behave as a *bona fide* terminal locus since it keeps a low Ci and segregates early (compare Figure 5B with Figures 4C and D). Its presence in the termination zone is however associated with a tendency to remain in the central third of the cell, even in cells with two foci (Figure 5C). Its positioning thus tends to resemble that of *ydeQ* in XL151 *tus*+: intermediate between the quarters and the midcell positions. On the other hand, the *ydeQ* locus is far from the termination zone and replicated early compared to termination in the restored terminus of the *tus*- derivative of XL151 (Figure 5A). This enhances its positioning toward the quarter positions (Figure 5B and C). However, it keeps a high Ci and segregates later than any other locus far from the termination zone (compare Figure 5B with Figure 4). We conclude that restoration of the termination zone opposite *oriC* in XL151 does not suppress the segregation/positioning defects of the different loci. Thus, although the replication timing of a locus compared to termination is an important determinant of its segregation, it is not the only one. This is clear for the PJ region (the *ydeQ* locus), which keeps some features of a terminal locus, particularly a high Ci, independently of its replication timing. The replication timing rather influences the home positioning of loci after their segregation (Figure 5C). The termination zone tends to remain in the middle third of the cell whereas loci located along the replichore migrate towards quarter positions.

### FtsK Is Essential in XL151

Since XL151 displays defects in post-replicative segregation and cell division, we tested the effect of two factors that are involved in coupling segregation and cell division, FtsK and SlmA. SlmA is a nucleoïd-associated protein that prevents divisome assembly in nucleoïd-containing zones [37]. Inactivation of SlmA in XL151 had little if any effect on cell morphology (not shown) and on doubling time in permissive conditions (Table 2). This is consistent with the observation that most double-cells in XL151 populations harbour a constricting septum, suggesting that division is delayed or blocked after assembly of the divisome.

**Table 2:** Doubling time of XL151 and derivatives.

Strain	Doubling time (minutes)
Wild type	64
Wild type *ftsK*_ATP-_ P_BAD_-*ftsK*_wt_ Ara 0,2%	65
Wild type *ftsK*_ATP-_ P_BAD_-*ftsK*_wt_ Glu 0,2%	75
XL151	92
XL151 *ftsK*_ATP-_ P_BAD_-*ftsK*_wt_ Ara 0,2%	94
XL151 *ftsK*_ATP-_ P_BAD_-*ftsK*_wt_ Glu 0,2%	NA
XL151 *xerC^-^*	100
XL151 *tus^-^*	86
XL151 *slmA^-^*	91

NA: not applicable.

**Figure 6:**
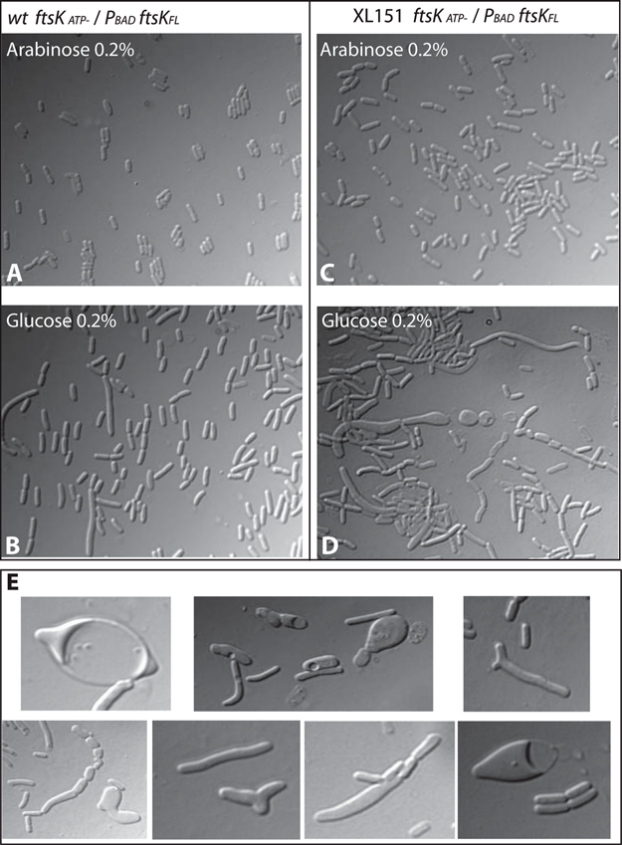
Depletion of FtsK in XL151. Depletion of FtsK was done in wt *ftsK_ATP-_* and XL151 *ftsK_ATP_*_-_ strains containing a P_BAD_-*ftsK_wt_* allele on a plasmid. (A and B): Growth in presence of arabinose (0,2%) allows complementation of the *ftsK_ATP-_* phenotype in both the wt *ftsK_ATP-_* (A) and XL151 *ftsK_ATP_*_-_ (B) strains. (C and D): Depletion of FtsK_wt_ was achieved by growing the cells 3 hours in liquid minimal medium containing 0.2% glucose at 30°C before microscopic observation. (C): wt *ftsK_ATP-_*; (D) XL151 *ftsK_ATP_*_-_. (E) Compilation of aberrantly shaped cells observed in XL151 *ftsK_ATP_*_-_ depleted for FtsK_wt_.

To assay the role of the translocase activity of FtsK, we used the *ftsK_ATP_*-Cm allele, which is defective in translocation and the *ftsKD(g)* allele which translocates with no orientation control [38],[39]. Since we were unable to transfer these allele into XL151, the strain was first transformed with a plasmid carrying an arabinose-inducible *ftsKwt* gene (Materials and methods). Both alleles were established in this strain only in presence of arabinose and substituting arabinose for glucose or IPTG did not support growth, showing that the oriented translocase activity of FtsK is essential in XL151 (Figure S3). Importantly, the XerC recombinase is not essential in XL151 (Table 2; Figure S3), showing that the translocase activity of FtsK is required for a function other than activation of chromosome dimer resolution. As shown in Figure 6, the FtsK_ATP_^-^ phenotype in wt was suppressed in presence of 0.2% arabinose. Depletion of FtsK was then achieved by switching off *ftsKwt* expression for 3 hours. Microscopic observation revealed numerous cells with aberrant morphology (Figure 6). Not only did cells form filaments and chains as usually seen with *ftsK* mutants defective in translocation [39], but also abnormally wide cells, Y-shaped cells, lemon-shaped cells, mini-cells and spherical cells (Figure 6D and E). Time lapse microscopy was used to observe the formation and fate of aberrant cells (Movies S1 and S2). Cells were plated on minimal medium containing glucose after 3 hours growth under depletion conditions in liquid medium and photographed every 5 mn over a 5 hour period. Numerous cells form intra-cytoplasmic vesicles and lyse suddenly. These forms and fate are usually inferred to be due to defects in peptidoglycan synthesis, as in mutants of the PBP proteins [40],[41].

## Discussion

### An Original Cell Cycle Defect that Renders Translocation by FtsK Essential for Viability

Here we have shown that a perturbation of the replichore polarity perturbs the late steps of the cell cycle resulting in blockage of cell division and elongation. This phenotype is specific to strains carrying large chromosome inversions from the terminal junction of replichore polarity (PJ), which lead to a major imbalance of the two replichores. In such strains, the replication period (C) increases as a consequence of the lengthening of one replication arm. The post-replicative period (D) is lengthened or blocked depending on growth conditions, accounting for the accumulation of cells arrested at a late step of the cell cycle. These cells harbour apparently segregated nucleoïds and most often a constricting division septum, indicating that the blocked step is in late D period near the time of cell separation. Importantly, restoring termination of replication to the region opposite *oriC* suppresses the lengthening of the C period but does not suppress the division defect, suggesting that the late D period blockage is due to the unbalance of replichore polarity better than to the unbalanced replication.

A striking feature of strains carrying large inversions from the terminal PJ is that cell elongation is delayed or blocked at the same time as cell division. This phenotype is in contrasts to cell division phenotypes reported previously. When inhibition directly targets divisome components, with conditional mutants [42] or cephalexin [43], cell elongation and chromosome replication carry on, leading to filamentous cells. An equivalent phenotype is observed when division is inhibited owing to segregation defects, as in nucleoïd occlusion mediated by the SlmA protein [37] or decatenation defects due to TopoIV inactivation [44]. In other cases, segregation defects do not directly inhibit cell division, but are accompanied by chromosome damage leading to SOS-induced filamentation (e.g., in the case of unresolved chromosome dimers [45]). Contrary to these cases, the division blockage reported here occurs without major deregulation of the cell cycle. Initiation of replication occurs normally once per cycle and stops in blocked cells, possibly as a consequence of the elongation stop. Few filaments or other abnormal cells form, indicating that these outcomes are not the normal fate of blocked cells. In addition, most cells survive and can form colonies even after several hours of blockage. It thus appears that cells suspend all steps of their cycle waiting for division to proceed, which reveals a point of control not previously described to our knowledge.

### The Oriented Translocation Activity of FtsK Is Essential in XL151

The KOPS-directed DNA translocase activity of the FtsK protein becomes essential for growth after inversion, whereas it is mainly required when a chromosome dimer has formed in the wt strain [46]. The essentiality of FtsK translocation in XL151 is not due to an elevated frequency of chromosome dimers since inactivation of XerC only has a moderate effect on viability. Chromosome processing in the vicinity of the division septum is thus strictly required in XL151 independently of the monomeric or dimeric state of the chromosomes. Notably, the FtsK translocase domain is also essential for growth in *mukB*- strains in which the structure and/or segregation of the chromosome is affected [47]. In XL151, segregation of the PJ is affected and neither of the inversion endpoint segregates like the wt PJ. However, we have previously shown that in XL151 and equivalent strains FtsK can reach the PJ since it translocates to the *dif* site to resolve chromosome dimers [33].

We assume that FtsK action is required and/or slowed by the displacement of the PJ and that this, in turn, slows or blocks the closure of the division septum. It follows that septum closure depends on a correct processing of the replichore junction by FtsK, independently of the presence of chromosome dimers. In this view, non-permissive conditions, which accelerate the C and D periods, may not allow sufficient time for FtsK to reach the PJ before the end of the next replication cycle that may counteract FtsK translocation to the PJ. We propose that cell division is controlled by FtsK translocation to the PJ in strains harbouring asymmetric replichores. Whether or not this control operates in wt strains remains to be shown.

### A Role for FtsK in the Control of Membrane Synthesis

Our data strongly suggest that the translocase activity of FtsK is involved in the control of membrane synthesis in inversion-carrying strains. Depletion of FtsK in an *ftsK_ATP_* mutant of strain XL151 provokes the appearance of aberrant cell morphologies reminiscent of those due to defects in peptidoglycan synthesis [40],[41]. Interestingly, possible relationships between FtsK and peptidoglycan synthesis have already been reported. A temperature sensitive allele of *ftsK*, *ftsK_44_*, is suppressed by inactivation of DacA (PBP5) [23], a penicilin-binding protein implicated in cell shape maintenance [41]; and FtsK interacts with FtsI (PBP3) [48]. Both PBP3 and PBP5 are involved in peptidoglycan synthesis at the septum. This reinforces the view that FtsK translocation to the PJ may control late steps of septum synthesis. The fact that FtsK interacts with FtsI via its C-terminal militates towards this hypothesis [49]. The FtsK-dependent control of septum synthesis may in turn participate in the control of cell elongation since cell elongation and cell division are alternating and competing events in *E. coli* [50] and *B.subtilis* 51,52.

### Roles of Replication and Replichore Polarity in Segregation of the Rearranged Chromosome

Analysis of the intracellular positioning of chosen loci in wt and XL151 reveals different roles for the proximity with the replichore polarity junction (PJ) and the termination zone in their segregation pattern. Loci neighbouring the PJ in the wt strain remain colocalised (cohesive) long after their replication. This does not depend on the timing of their replication since the PJ keeps a high colocalisation index (Ci) after inversion independently of its replication timing. In contrast, loci far from the PJ in both the wt and inverted strain displays a low Ci, even when present close to the termination zone. Taken together, these data strongly suggest that sister-chromatid cohesion at a given locus is not controlled by its replication timing but more likely due to its proximity with the PJ. Termination of replication nevertheless has a strong influence on segregation of neighbouring loci but affects the positioning of loci after their migration rather than their colocalisation time. Loci close to the termination zone tend to remain in the middle third of the cell whereas those located along the replichore migrate towards quarter positions. This is consistent with the proposal that the timing of replication of a locus determine its home position after migration [5].

## Materials and Methods

### General Procedures and Constructs

Bacterial strains were grown in L broth or M9 broth (0,2% casamino acids; 0,4% glucose). Ampicilin (25g/ml), kanamycin (50g/ml), streptomycin (200g/ml), gentamycin (7.5g/ml) and chloramphenicol (15g/ml) were added when required. The wild type strain (wt) is derived from XL13 (MG1655 Δ*lac_Mlu_*_I_ Δ*attB*::Sp/Sm *zdd347::lacZ::attB rpsL* (StR); [33]). Strains carrying the Inv(*dif-sp5*), Inv(*dif-ydfE*) and Inv(*dif-sp39*) inversions keeping *dif* at the replichore polarity junction were previously described [33]. The *ftsK_ATP_-*Cm [39], and *xerC*::Gm alleles [26] were transferred by P1 transduction. The *slmA*::Tc and *tus*::Tc mutations were constructed using standard techniques, inserted on the chromosome using integration-excision vectors and transferred by P1 transduction. Plasmid pFX465 carrying an arabinose-inducible *ftsK* allele was kindly given by FX Barre. The *parS*-Cm fragment from pGBKD3-*parS* was inserted on the chromosome at positions 4,413,507 (*aidB*), 257,144 (*crl*), 1,584,764 (*ydeQ*) and 1,596,522 (*ydeV*) using standard in vitro constructs and the “lambda red” technique [53]. The D30ParB-GFP fusion protein was expressed from plasmid pALA2705 [9]. Live/Dead staining was perform using the BacLight kit.

### Flow Cytometry

Flow cytometry was performed as described in [54]. For origin counting, samples were prepared after incubation with cephalexin and rifampicin for 3 hours, during which the current replication cycles terminate. This provide a snapshot of the number of replication origin per cell at the time of drugs addition (Table S2) [55]. For measurement of the C period, sample were prepared every 10 min after addition of rifampicin. Analysis of the DNA content per cell were performed with a Win-Bryte flow cytometer from Bio-Rad. Calibrations for positions corresponding to one, two, etc. chromosome equivalents were carried out with stationary-phase cells and with cells subjected to rifampicin-replication run out. The flow-cytometry data files were transferred to FCSPRESS.55b (Power Macintosh) and Flowjo software (www.flowjo.com) for plotting and analysis.

### Microscopy

Cells carrying a *parS* insertion and plasmid pALA2705 were grown at 30°C in M9 broth (0,2% casamino acids; 0,4% glucose) without IPTG (the D30ParB-GFP protein was produced from the basal level of the *lacZp* promoter) to OD_600_ = 0,3, concentrated 50 fold by centrifugation and placed on a polylysine-coated glass slide. Microscopy was carried out on a Zeiss upright axioplan II microscope. Images were analysed using the Metamorph software.

### Calculation of the Colocalisation Index (Ci)

The co-localisation index is the mean copy number of a locus calculated from flow cytometry data divided by its mean number of foci per cell. Detail are in Sup.Table 3.

## Supporting Information

Figure S1 Duration of the C period in wild type and XL151 strains.
(1.12 MB TIF)

Figure S2 Cell cycle of the wild type (A) and the XL151 (B) strains.
(0.69 MB TIF)

Figure S3 The oriented translocation activity of FtsK is essential in XL151.
(4.80 MB TIF)

Table S1 Cell size distribution of the different strains in different growth conditions analysed by flow cytometry.
(0.03 MB DOC)

Table S2 Quantification of the number of replication origin per cell.
(0.04 MB DOC)

Table S3 Calculation of the Colocalisation index (Ci).
(0.04 MB DOC)

Video S1 Depletion of FtsK in XL151. XL151 ftsK(ATP-)/pFtsKwt cells were plated on minimal medium containing glucose after 3 hours growth under depletion conditions in liquid medium and photographed every 5 mn over a 5 hour period.
(4.32 MB MOV)

Video S2 Depletion of FtsK in XL151. XL151 ftsK(ATP-)/pFtsKwt cells were plated on minimal medium containing glucose after 3 hours growth under depletion conditions in liquid medium and photographed every 5 mn over a 5 hour period.
(3.20 MB MOV)
