# Supplementary figures and images for: Asymmetry of Chromosome Replichores Renders the DNA Translocase Activity of FtsK Essential for Cell Division and Cell Shape Maintenance in Escherichia coli
Source: PLoS Genet. 2008 Dec 5;4(12):e1000288. doi: 10.1371/journal.pgen.1000288 (PMC2585057; doi:10.1371/journal.pgen.1000288)

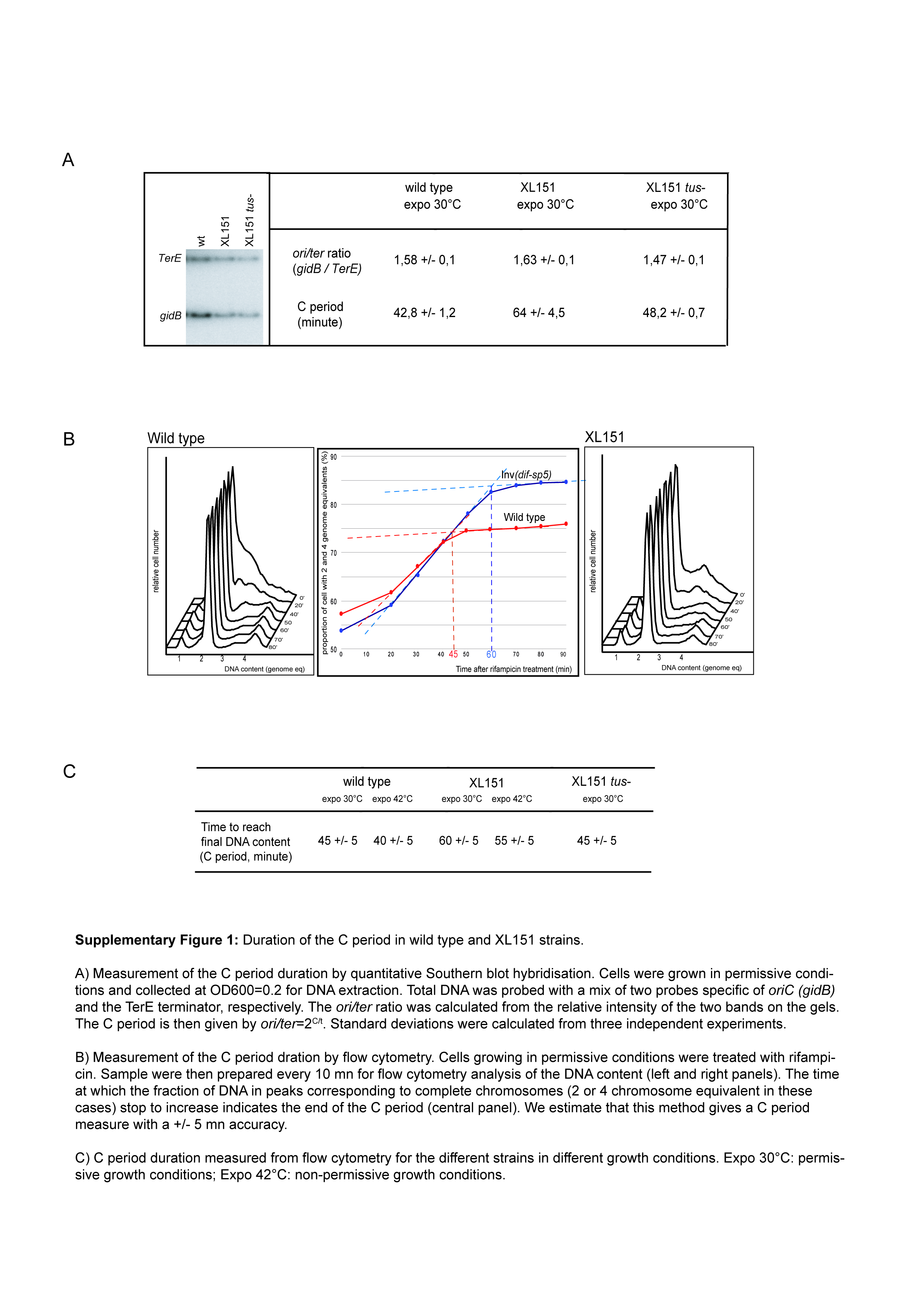

Supplement: Figure S1 — Duration of the C period in wild type and XL151 strains. (1.12 MB TIF) [file pgen.1000288.s001.tif]

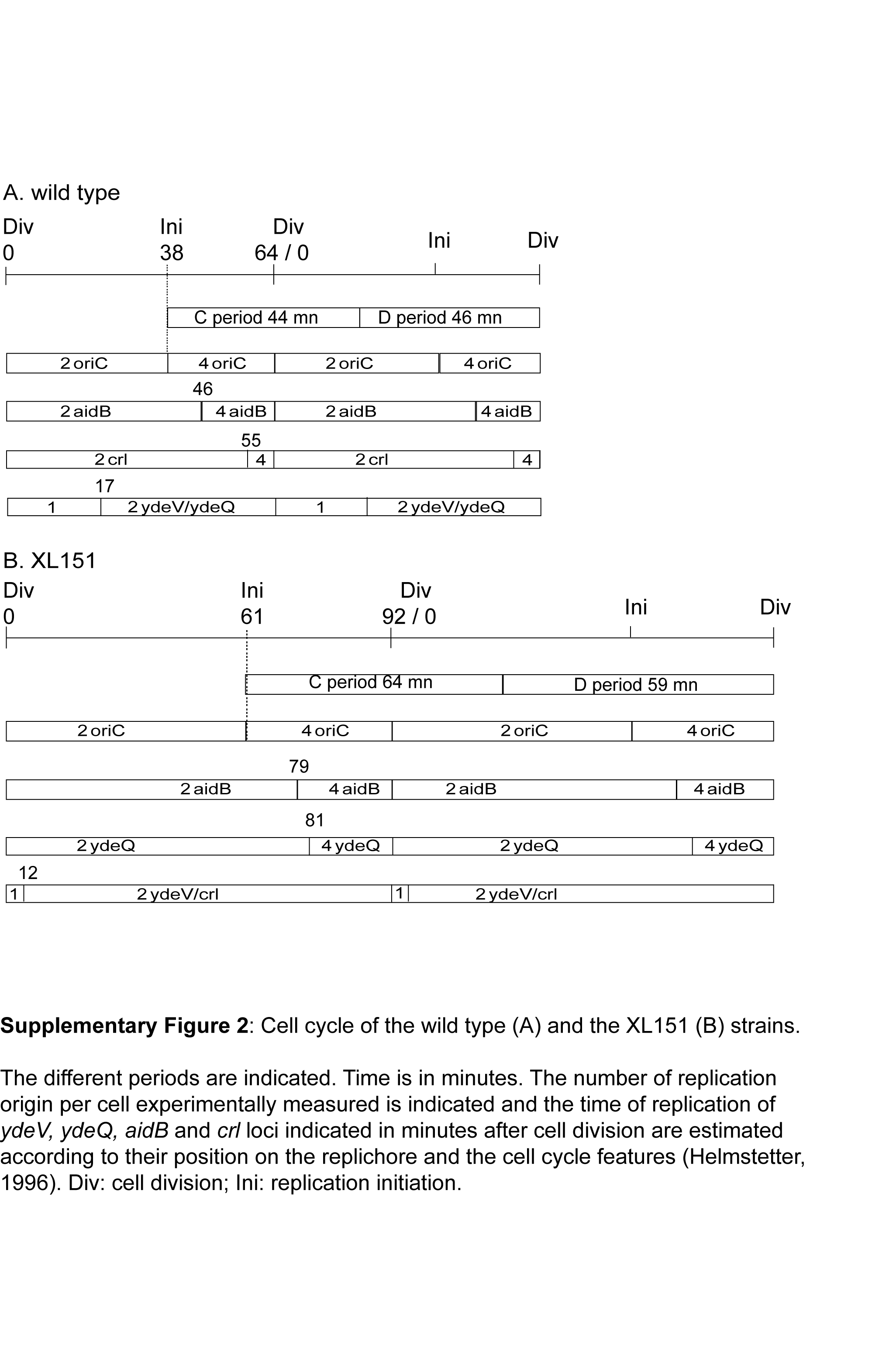

Supplement: Figure S2 — Cell cycle of the wild type (A) and the XL151 (B) strains. (0.69 MB TIF) [file pgen.1000288.s002.tif]

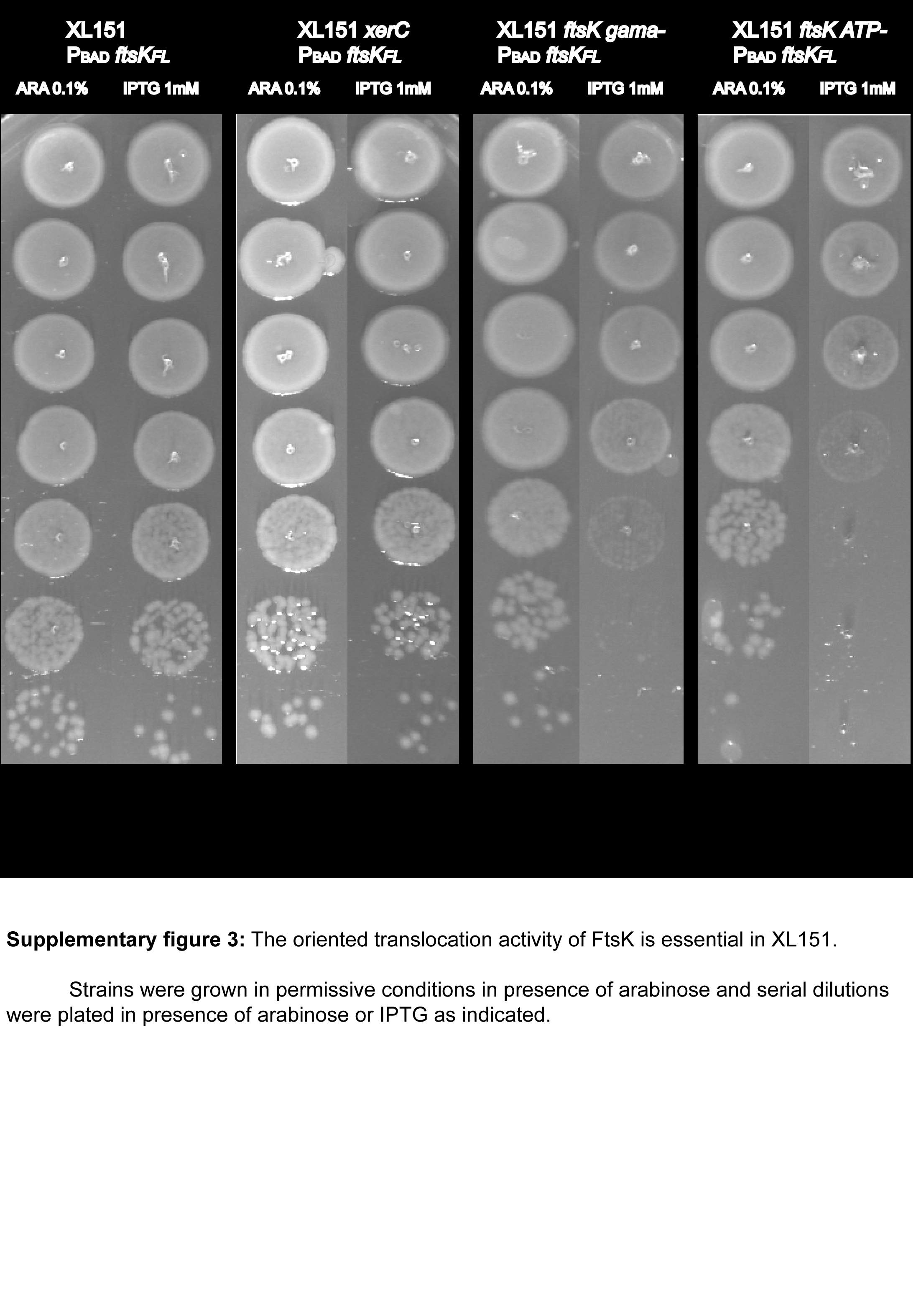

Supplement: Figure S3 — The oriented translocation activity of FtsK is essential in XL151. (4.80 MB TIF) [file pgen.1000288.s003.tif]
