# Supplementary material for: Asymmetry of Chromosome Replichores Renders the DNA Translocase Activity of FtsK Essential for Cell Division and Cell Shape Maintenance in Escherichia coli
Source: PLoS Genet. 2008 Dec 5;4(12):e1000288. doi: 10.1371/journal.pgen.1000288 (PMC2585057; doi:10.1371/journal.pgen.1000288)
Supplement: Table S1 — Cell size distribution of the different strains in different growth conditions analysed by flow cytometry. (0.03 MB DOC) [file pgen.1000288.s004.doc]

| Cell size (µm) | <4 | 4-7 | >7 |
| --- | --- | --- | --- |
| wild type  Stat  Growth 30°C  1H 42°C | 95  81  86 | 5  18  14 | <1  <1  <1 |
| XL151  Inv(dif-sp5)  Stat  Growth 30°C  1H 42°C | 96  36,5  19 | 4  58  78 | <1  5,5  3 |
| Inv(dif-ydfE)  Stat  Growth 30°C  1H 42°C | 96  74  79 | 3  21  16 | <1  5  5 |
| Inv(dif-sp39)  Stat  Growth 30°C  1H 42°C | 97  44  20 | 2  53  75 | <1  3  5 |

**Supplementary Table 1:** Cell size distribution of the different strains in different growth conditions analysed by flow cytometry.

Cell were prepared for flow cytometry analysis in three growth conditions: after overnight growth in permissive conditions (stationary phase, Stat); in exponential growth in permissive conditions (growth 30°C); after one hour incubation in non-permissive conditions (1h 42°C). Quantification of subpopulations of cell (given in % of the total population) under 4µm, from 4µm to 7µm and over 7µm are presented for the wt strain, the XL151 (Inv(*dif-sp5*)) strain and two previously described strains carrying inversions from the terminal replichore polarity junction (Lesterlin *et al*., 2005): one carrying a 800kb Inv*(dif-ydfE)* inversion that provokes a phenotype equivalent to the XL151 phenotype, the other carrying a 59,5 kb Inv(*dif-sp39*) inversion that do not provoke over-representation of dividing cells.
