# Supplementary material for: Asymmetry of Chromosome Replichores Renders the DNA Translocase Activity of FtsK Essential for Cell Division and Cell Shape Maintenance in Escherichia coli
Source: PLoS Genet. 2008 Dec 5;4(12):e1000288. doi: 10.1371/journal.pgen.1000288 (PMC2585057; doi:10.1371/journal.pgen.1000288)
Supplement: Table S2 — Quantification of the number of replication origin per cell. (0.04 MB DOC) [file pgen.1000288.s005.doc]

| % of total cells | Cell with  1 origin (o1) % | Cell with  2 origins (o2) % | Cell with  3 origins (o3) % | Cell with  4 origins (o4) % | Origins per cell ratio  (No) |
| --- | --- | --- | --- | --- | --- |
| wild type  Stat  Growth 30°C  1H 42°C | 58  2  0,3 | 37  61  65 | 3  4  2,7 | 2  33  32 | 1,49  2,68  2,66 |
| XL151  Inv(dif-sp5)  Stat  Growth 30°C  1H 42°C | 75  2  0,5 | 24,5  68  42,5 | 0,4  3  2,5 | 0,1  27  54,5 | 1,25  2,55  3,11 |
| Inv(dif-ydfE)  Stat  Growth 30°C  1H 42°C | 51,2  3,5  3 | 45  52,5  65 | 1,8  3,5  3,5 | 2  40,5  28,5 | 1,54  2,81  2,57 |
| Inv(dif-sp39)  Stat  Growth 30°C  1H 42°C | 71,5  2,5  0,5 | 27,8  65,5  49,2 | 0,5  4,2  2 | 0,2  27,7  48,3 | 1,29  2,57  2,98 |
| XL151 tus-  Inv(dif-sp5)tus-  Stat  Growth 30°C | 77  3 | 23  71 | 0  3,5 | 1  22,5 | 1,27  2,45 |

Supplementary Table 2: Quantification of the number of replication origin per cell.

The origins per cell ratio (No) is given by No= (1 x o1)+(2 x o2)+(3 x o3)+(4 x o4)/100 where o1, o2, o3, and o4 are the ratio of cells with respectively 1, 2 3 and 4 origins measured by the cephalexin-rifampicine run out experiment presented in figure 3. Strains and growth conditions: see Table S1 for legend.
