# Supplementary material for: Asymmetry of Chromosome Replichores Renders the DNA Translocase Activity of FtsK Essential for Cell Division and Cell Shape Maintenance in Escherichia coli
Source: PLoS Genet. 2008 Dec 5;4(12):e1000288. doi: 10.1371/journal.pgen.1000288 (PMC2585057; doi:10.1371/journal.pgen.1000288)
Supplement: Table S3 — Calculation of the Colocalisation index (Ci). (0.04 MB DOC) [file pgen.1000288.s006.doc]

| **Wild type** | | | |
| --- | --- | --- | --- |
| Locus name  and position (ratio of the replichore length) | Locus copy number per cell  Replication timing Rt  (ratio of the generation time) | Mean number of visible foci per cell | Colocalisation index Ci (Locus copy number / Number of foci per cell) |
| *aidB*  21% | 2.5  Rt = 74.4 % | 1.81  (29 ;61 ;10 ;0) | 1.38 |
| *Crl*  40% | 2.25  Rt = 87.5% | 1.7  (35 ;59 ;4 ;2) | 1.32 |
| *ydeV*  28% | 1.72  Rt = 28% | 1  (98 ;2 ;0 ;0) | 1.72 |
| *ydeQ*  98.4% | 1.72  Rt = 28% | 1  (99 ;1 ;0 ;0) | 1.72 |

| **Inv(*dif-sp5*)** | | | |
| --- | --- | --- | --- |
| Locus name  and position (ratio of the replichore length) | Locus copy number per cell  Replication timing Rt  (ratio of the generation time) | Mean number of visible foci per cell  (f1; f2; f3; f4) | Colocalisation index Ci (Locus copy number / Number of foci per cell) |
| *aidB*  21% | 2.3  Rt = 85% | 2.04  (10 ;80 ;6 ;4) | 1.12 |
| *Crl*  63% | 1.87  Rt = 12.8% | 1.61  (48 ;45 ;5 ;2) | 1.16 |
| *ydeV*  62% | 1.88  Rt = 12% | 1.59  (57 ;30 ;10 ;3) | 1.18 |
| *ydeQ*  25% | 2.24  Rt = 88% | 1.37  (63 ;37 ;0 ;0) | 1.63 |

**Supplementary Table 3: Calculation of the Colocalisation index (Ci)**

The Ci is given by: Ci = (mean locus copy number) / (mean number of distinguishable fluorescent foci)

The mean number of distinguishable fluorescent foci is calculated with the ratio of cells with 1 focus, 2, 3 or 4 foci indicated by (f1;f2;f3;f4), and equals

Mean foci number = ((1xf1) + (2xf2) + (3xf3) + (4xf4)) / 100

The mean copy number of a locus depends on its replication timing Rt (Helmstetter, 1996).

Rt = ((2t-(C+D))+xC) / t

C:Cperiod; D:Dperiod; t: generation time; x position of the locus as a ratio of the replication arm.
